# Effects of Oral Lipid‐Based nutritional supplements on appetite, energy intake, and lipid profile of moderately underweight children

**DOI:** 10.1002/fsn3.3125

**Published:** 2022-12-18

**Authors:** Aqsa Zubair, Sadia Fatima, Hamid Habib, Rubina Nazli, Inayat Shah, Mohsin Shah

**Affiliations:** ^1^ Department of Biochemistry Institute of Basic Medical Sciences (IBMS), Khyber Medical University (KMU) Peshawar Pakistan; ^2^ Department of Physiology Institute of Basic Medical Sciences (IBMS), Khyber Medical University (KMU) Peshawar Pakistan; ^3^ Department of IBMS Peshawar Pakistan

**Keywords:** appetite, BMI, energy intake, lipid profile, LNS, malnourishment, total cholesterol

## Abstract

Oral lipid‐based nutritional supplements (LNS) are designed to ensure dietary adequacy and to improve malnourishment in children. Therefore, this study investigated the effects of 4 weeks of LNS on appetite, energy intake, and lipid profile of moderately underweight children (5–10 years old) with BMI‐Z score between −2 and − 3 SDS, recruited in a single‐blind randomized control trial. In addition to the regular dietary intake, fasting blood samples, anthropometric measurements, energy intake, and appetite responses were obtained before and after 4 weeks of LNS (535 kcal) or PLACEBO (92 kcal). After 4 weeks of supplementation mean energy intake (kcal) (*p* < .001), body weight (kg) (*p* < .001), BMI (kg/m^2^) (*p* < .01), mid‐upper arm circumference (cm) (*p* < .01), total cholesterol (mg/dl) (*p* < .01) and fasting glucose (mg/dl) (*p* < .01) were raised significantly in the LNS group as compared to the PLACEBO group. No significant changes were detected in appetite responses (p > 0.05). In conclusion, LNS increases the overall energy intake, but does not affect the appetite but may induce hyperglycemia and hyperlipidemia.

## Graphical abstract

This trial report the effects of lipid‐based nutritional supplements (LNS) on moderate underweight school going children. The recruited children (both girls and boys) with mean age 7.1 ± 1.5 years had a BMI‐Z score between ‐2 and ‐3 SDS. We found a significant increase in weight, BMI, mid upper arm circumference, energy intake, total cholesterol and fasting glucose in LNS group. Data from this study can be used in future programming of LNS for improvement of malnourished status in children.

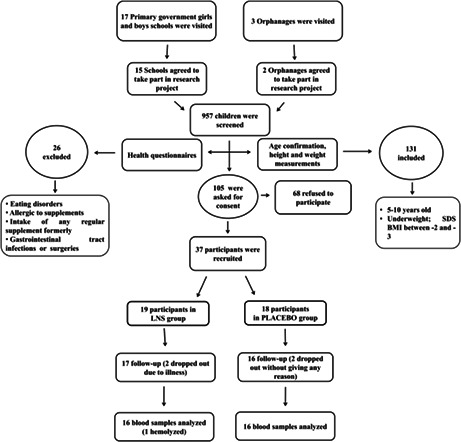

## 1 INTRODUCTION

Malnutrition is a pathological condition that may occur due to either excess or deficiency of micronutrients, macronutrients, or energy intake (Rahman & Hakim, 2015). In developing countries, about 50 million children are wasted, 183 million are underweight, and 156 million are stunted (Khara et al., 2018). About 35% of children deaths in Pakistan are attributed to malnutrition having nine times greater mortality risk (Akhtar et al., 2013). In 2018, the National nutritional survey of Pakistan indicated 28.9% of children under the age of 5 were underweight, 40.2% stunted and 17.7% were wasted (UNICEF, n.d.).

Childhood malnutrition is correlated with illiteracy, poor hygiene (Black et al., 2013), unbalanced diet, poverty (Juma et al., 2016), socioeconomic conditions, and diseases (Khan et al., 2016). Similarly, household food insecurity, food taboos, population pressure, gender bias, and man‐made disasters are a few other reasons behind malnutrition (Hassan et al., 2016). In Pakistan, most of the children are either partially immunized or not immunized against childhood diseases, making them vulnerable to infections (Imdad et al., 2011) and malnutrition (Hirani, 2012). Poor breastfeeding practices along with low quality of complementary foods also contribute to malnutrition (Mujib et al., 2006).

Among the strategies to revive moderately acute malnourished (MAM) children, the most acceptable regime is the use of LNS (World Health Organization, 2007). A randomized controlled trial conducted by Huybregts et al., observed significant effects on the growth rate of undernourished children after 4 months of LNS (Huybregts et al., 2012). Another study found 29% increase in recovery rate and a significant improvement in weight‐for‐height in MAM children provided with nutritional supplements (Lazzerini et al., 2013). Similarly, a cluster‐randomized study on infants observed that a supplementation of 500 kcal ready‐to‐use therapeutic food (RTUF) per day decreased the chances of wasting over a follow‐up period of 8 months (Isanaka et al., 2009). Similarly, a 4 months′ follow‐up non‐randomized cohort study on 2238 children observed a positive effect on anthropometric measurements and decreased prevalence of wasting by taking ready‐to‐use supplementary food (RUSF) (Grellety et al., 2012). Similar findings were observed in severely acute malnourished (SAM) and MAM children where the recovery rate was enhanced during supplementation period (Defourny et al., 2009; Karakochuk et al., 2012). In contrast, a study found lower than expected weight gain in underweight children after the intake of LNS (Fatima et al., 2018). Concomitantly, in lean women LNS suppressed the appetite and energy intake after the use of LNS (Fatima, et al., 2015).

Appetite is a physiological process that influences energy intake and is associated with motivational states like hunger, fullness, satiety, and desire to eat (Gibbons et al., 2019). Poor appetite causes insufficient food intake which in turn causes undernutrition in children (Waterlow, 1994). A study found that imbalance in hormones like leptin and insulin may also cause loss of appetite (Arsenault et al., 2007). It was also observed that the use of LNS may reduce the insulin sensitivity (Fatima, Gerasimidis, Wright, & Malkova, 2015). Undernourished people often suffer from dyslipidemia (Lumey et al., 2009) however, no effects on fasting lipids were observed after using LNS for 5 days (Fatima, et al., 2015).

Previous studies have documented the impact of various multi‐micronutrient supplements on the improvement of appetite in stunted and undernourished children (Dossa et al., 2002; Shakur et al., 2009; Stoltzfus et al., 2004). However, no clinical trial has been conducted to measure the changes in appetite of MAM children. Since the effectiveness of LNS interventions for the prevention of undernutrition is rapidly increasing (Ashorn et al., 2013), there is noticeable lack of evidence on possible effect of LNS on appetite of MAM children. Many targeted interventional programs have been implemented to improve energy intake in underweight children. However, the effect of LNS on energy intake and appetite in MAM children is scarce. Similarly, the efficacy of LNS on lipid profile has been demonstrated in many studies in adults and obese children but the data on effects of LNS on lipid profile in MAM children is hitherto unknown. Therefore, this study was designed in the community set‐up for school‐going MAM children to investigate the effect of LNS on appetite. In addition, changes in glucose, lipid profile, insulin, and energy intake were explored.

## 2 MATERIALS AND METHODS

### 2.1 Study design

**FIGURE 1:**
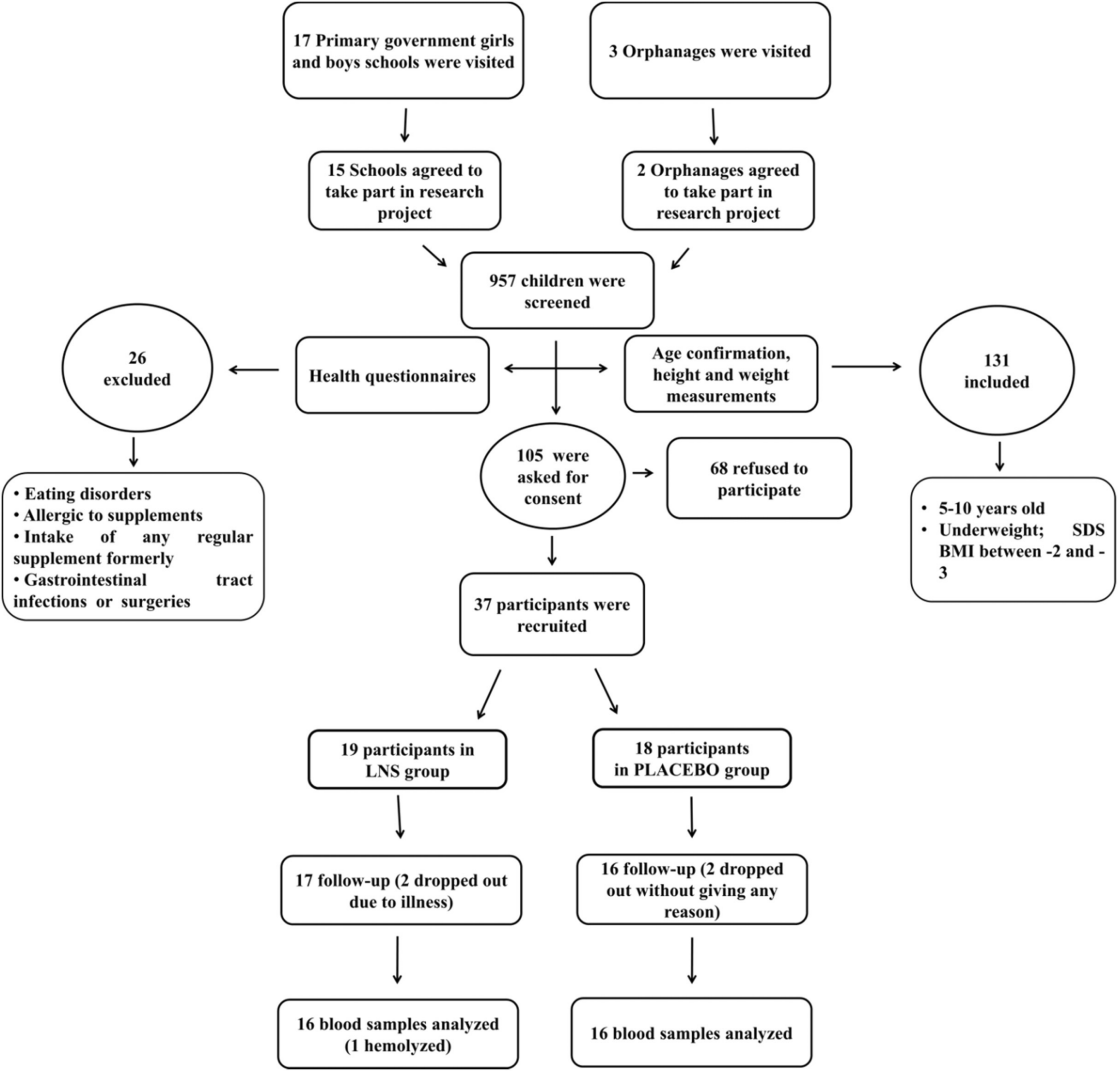
Schematic representation of participants' recruitment and follow‐up during the trial

This single‐blind randomized control study was carried out at clinical trial room, IBMS, KMU, and 12 public sector schools and orphanages of Peshawar, from September, 2017 to April, 2018. The study was approved by the Ethical Board of KMU (Study approval No. DIR/KMUEB/EN/000454). Children were approached after gaining permission from schools' administrations. Children with any food or medicine allergies, eating or gastrointestinal disorders were excluded. After taking the anthropometric measurements according to WHO standard procedures, 37 school‐going mild‐to‐moderate underweight children (BMI Z‐score between −2 and − 3 SDS) between 5 and 10 years of age were selected. Written as well as verbal informed consent from the guardians (parents) and assent from children were obtained. The children (20 boys and 17 girls) were randomly allocated into two groups using Computer Randomizer software into the LNS group (*n* = 19) and the PLACEBO group (*n* = 18). This sample size was calculated using online software “Openepi.” The data to calculate this sample size were obtained from a previous study by Fatima et al., (Fatima, et al., 2015). This sample size was enough to detect a difference of approx. 535 kJ of energy intake with 80% power at level of 5% to spot a difference of around two‐thirds of the standard deviation between the LNS and the PLACEBO for any measure (Figure 1). The LNS used in this study was “Acha Mum” by WFP (World Food Programme) providing 535 kcal/day (Table 1). The PLACEBO was prepared with 42 g roasted chickpeas (68 kcal) and 58 g low‐fat milk (Nesvita; 24 kcal). It provided 92 kcal/day energy (Table 1). No conflict of interests was found among any group involved in this study.

**TABLE 1:** Composition of LNS and PLACEBO

Sr. no.	Components	LNS (100 g/day)	PLACEBO (100 g/day)
1.	Energy (kcal)	535	92
2.	Energy (kJ)	2238	384.6
	Macronutrients (g)		
3.	Proteins	14	5.54
4.	Fats	30	0.58
5.	Carbohydrates	53	16
6.	Omega‐3 polyunsaturated fatty acids (% TE)	1.6	0
7.	Omega‐6 polyunsaturated fatty acids (% TE)	7.8	0
	Minerals (mg)		
8.	Zinc	11	0
9.	Iron	10	0.22
10.	Phosphorus	450	1.74
11.	Potassium	900	0
12.	Calcium	535	0.16
13.	Vitamin D	15 mcg	0

Abbreviations: Lipid‐based nutritional supplement.

### 2.2 Socio‐demographic data

Socio‐demographic data were collected using pre‐designed proforma. Data regarding parents' characteristics (status, occupation, education, and monthly income), number of siblings and family members, family accommodation/expenditure, and housing quality were recorded from all the participants.

### 2.3 Main trial days (day 1st and day 31st)

**FIGURE 2:**
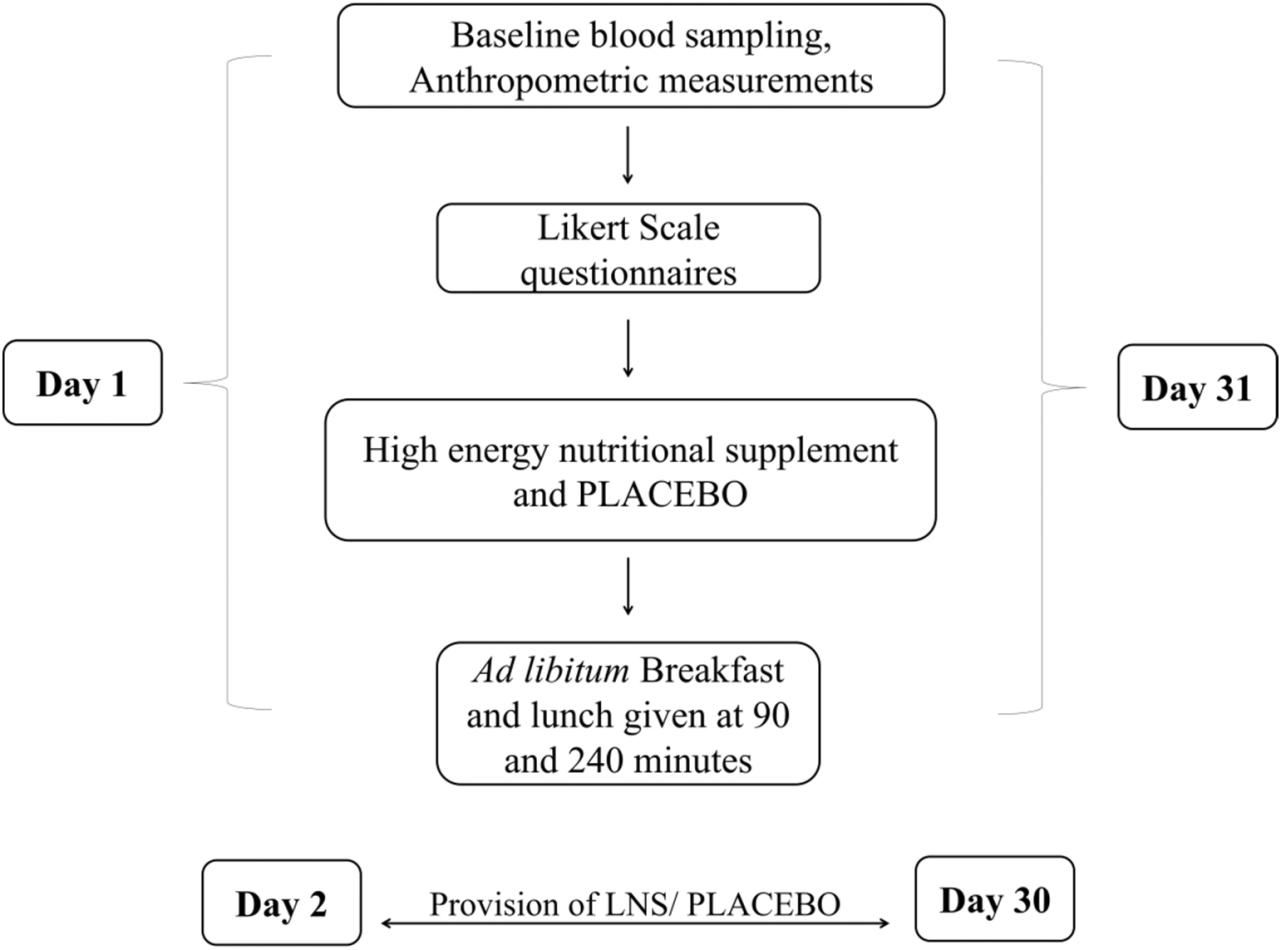
First and second trial day procedure

On main trial days, that is, day 1 and day 31, participants reported to the clinical trial room between 07:30 and 08:30 a.m. in fasting state. After taking fasting blood samples, anthropometric measurements were taken. It included body height, body weight‐for‐age, skinfold thickness, mid‐upper arm circumference (MUAC), and waist/hip ratio. Participants marked the appetite questionnaires at time point 0, that is, before the consumption of LNS or PLACEBO. The questionnaire was further marked at 30‐ and 60‐minute post supplementation, followed by an ad libitum buffet breakfast at 90 min. The questionnaire was again marked at 120, 150, 180, and 210 min, after the supplementation followed by lunch at 240 min. During the trial, LNS/PLACEBO supplements were provided to children at their school daily for 1 month and empty packets were collected back to check compliance (Figure 2).

### 2.4 Anthropometric measurements

Height of participants was measured by portable stadiometer using a stretch stature method to the nearest 0.1 cm. Weight was measured by using Beurer digital glass weight scale “GS 200 Allium”. Shakir's measuring tape was used to measure mid‐upper arm circumference (MUAC) on the non‐dominant hand to the nearest 0.1 cm. Skin‐fold measurements, that is, biceps, triceps, mid‐abdomen, and sub‐scapular thickness were checked using “Holtain skin‐fold caliper” to the nearest 0.2 mm. Waist‐to‐Hip ratio was obtained by taking the waist and hip measurements using “Lasso‐o measuring tape.”

### 2.5 Blood parameters

Blood samples collected in fasting state were analyzed for glucose, insulin, and lipid parameters, that is, total cholesterol (TC), triglyceride (TG), high‐density lipoproteins (HDL), and low‐density lipoproteins (LDL). After centrifugation, the blood serum was stored at −80°C. “Roche Cobas Immunoassay E411 Analyzer” was used to analyze insulin and glucose, while “Roche Cobas C501 Chemistry Analyzer” was used for lipid profile analysis.

### 2.6 Energy intake measurements

Dietary Windiet software 2005 was used to calculate energy intake. To enhance the accuracy of results, three individual researches noted the values and then mean value was taken as final result.

### 2.7 Multiple pass 24‐hour dietary recalls

Dietary data of the participants were collected using multiple pass 24‐hour dietary recalls. The data were collected three times for three consecutive days, that is, 3 days before the 1st trial day, 3 days after 1st trial, and 15 days after 1st trial day. This was done to check the effects of supplement on habitual dietary intake of participants. The difference between the regular intake of participants before and after the supplement was also measured and compared.

### 2.8 Appetite measurement

In this study, five‐point Likert scale appetite questionnaires were used to check the responses to assess hunger, satiety, fullness, appetence, and desire to eat. A Likert scale is a psychometric scale used in research that employs questionnaires (Norman, 2010). Each questionnaire included a set of five questions with five responses. The participants had to choose one response about hunger, satiety, fullness, appetence, and desire to eat. Initially, the questionnaires were marked by the participants in fasted state (at 0 min) and at 30 and 60 min after supplementation followed by breakfast. The questionnaires were marked in pre‐lunch period, that is, at 120‐, 150‐, 180‐, and 210‐min post‐supplementation.

### 2.9 Hedonic scales measurements

Participants and the parents were inquired separately about the taste and acceptability of LNS using hedonic scales. The parents were also asked about any kind of progression noticed by them in their child after 1 month of supplementation.

### 2.10 Statistical analysis

Data analysis was done using Minitab version 17. To check weight‐for‐age Z‐score (WAZ) score, a software LMS (Least mean squares) Growth was used. Normality of data was checked by Anderson Darling test. Categorical data were compared by Chi‐square test. Appetite questionnaires and lipid profile were checked by paired two samples t‐test. *p*‐Value < 0.05 was taken as significant.

## 3 RESULTS

In this trial, 37 mild‐to‐moderate underweight children with age range 5–10 years and BMI‐Z score between −2 and − 3 SDS were recruited. A total of 32 blood samples (18 boys and 14 girls) were successfully collected by the end of the trial (Figure 1) and were analyzed for variations in anthropometric parameters, energy intake, appetite, lipid profile, insulin, and glucose levels.

### 3.1 Socioeconomic profile

Majority of the children belonged to low‐income backgrounds (monthly income from PKR. 5000 to 35,000). The fathers were mostly drivers, guards, and laborers. Similarly, most of the mothers were housewives. The literacy rate in parents was low. Most fathers were undergraduate, whereas 25% of fathers in LNS and 18.75% in the PLACEBO group were illiterate. Similarly, 62.5% of mothers from LNS and 81.25% from the PLACEBO group were illiterate. Furthermore, household status showed that majority of the children from both groups were living in separate families, urban areas and were using community tape water (81.25% in LNS and 87.50% in PLACEBO) (Table 2).

**TABLE 2:** Sociodemographic data of participants from LNS and PLACEBO groups

	LNS (*n* = 16)	*p*‐Value	PLACEBO (*n* = 16)	*p*‐Value
	Count (%)		Count (%)	
Professional status of father				
Employed	16 (100)	1	15 (93.75)	1
Unemployed	0		1 (6.25)	
Father education				
Illiterate	4 (25)	0.25	3 (18.75)	0.632
Primary	4 (25)		1 (6.25)	
Middle	1 (6)		6 (37.50)	
SSC	1 (6)		4 (25.00)	
HSSC	3 (18)		0	
Graduate	3 (18)		1 (6.25)	
Post graduate	0		1 (6.25)	
Professional status of mother				
Employed	1 (6.25)	1	2 (12.50)	1
Unemployed	15 (93.75)		14 (87.50)	
Mother education				
Illiterate	10 (62.50)	0.108	13 (81.25)	0.746
Primary	2 (12.50)		1 (6.25)	
Middle	1 (6.25)		0	
SSC	2 (12.50)		2 (12.50)	
HSSC	0		0	
Graduate	0		0	
Post graduate	1(6.25)		0	
Total family members				
0–3	0	0.997	0	0.999
4–6	8 (50)		5 (31.25)	
7–9	6 (37.50)		8 (50.00)	
10–12	1 (6.25)		2 (12.50)	
>12	1 (6.25)		1 (6.25)	
No. of siblings				
0–3	7 (43.75)	0.899	4 (25.00)	1
4–6	8 (50.00)		10 (62.50)	
7–9	0		2 (12.50)	
10–12	0		0	
>12	1 (6.25)		0	
Monthly income				
5000–15,000	4(25.00)	0.993	8 (50.00)	0.961
16,000–25,000	4(25.00)		3 (18.75)	
26,000–35,000	8(50.00)		5 (31.25)	
Household				
Separate	12 (75)	1	14 (87.50)	1
Joint	4 (25)		2 (12.50)	
House type				
Bungalow	0	1	0	1
Apartment	1 (6.25)		2 (12.50)	
Town house	14 (87.50)		13 (81.25)	
Village house	1 (6.25)		1 (6.25)	
House structure				
Pakka	12 (75.00)	0.96	13 (81.25)	0.955
Kacha	1 (6.25)		0	
Semi Pakka	3 (18.75)		3 (18.75)	
Others	0		0	
House status				
Rented	8 (50.00)	1	10 (62.50)	0.997
Self	8 (50.00)		5 (31.25)	
Employer/Govt	0		1 (6.25)	
Rent payment				
Self	15 (93.75)	0.999	14(87.50)	0.998
Govt.	0		1 (6.25)	
Other	1 (6.25)		1 (6.25)	
No. of kitchen				
0–3	16 (100.0)	1	16 (100.0)	1
4–6	0		0	
7–9	0		0	

Abbreviations: Govt, Government; HSSC, Higher secondary school certificate; SSC, Secondary school certificate.

*p < .05; **p < .01; ***p < .001.

### 3.2 Anthropometric characteristics analysis

There was a significant improvement in body weight [from 17.67 ± 2.8 kg to 18.36 ± 3.1 kg (*p* < .001)], BMI [from 12.96 ± 0.33 kg/m^2^ to 13.28 ± 0.46 kg/m^2^ (*p* < .01)] and MUAC [from 14.76 ± 0.94 cm to 15.18 ± 0.84 cm (*p* < .01)] in the LNS group in comparison to the PLACEBO group after 4 weeks of supplementation (Table 3).

**TABLE 3:** Comparison of anthropometric measurements between first and second trial days of the LNS group and the PLACEBO group

Parameters	Before LNS	After LNS	*p*‐Value	Before PLACEBO	After PLACEBO	*p*‐Value
Age (years)	6.94 ± 1.3	7.27 ± 1.37	0.19	7.26 ± 1.7	7.4 ± 1.66	0.53
Body height (cm)	116.4 ± 8.8	117.15 ± 8.8	0.120	122.3 ± 9.8	122.5 ± 9.74	0.38
Body weight (kg)	17.67 ± 2.8	18.36 ± 3.1	<0.001***	19.51 ± 3.01	19.72 ± 3.5	0.39
BMI (kg/m^2^)	12.96 ± 0.33	13.28 ± 0.46	0.002**	12.92 ± 0.32	13.03 ± 0.72	0.55
SDS height	−0.98 ± 1.41	−0.98 ± 1.47	0.96	−0.09 ± 1.26	−0.15 ± 1.24	0.10
SDS weight	−2.19 ± 1.05	−1.99 ± 1.18	0.007**	−1.57 ± 0.85	−1.58 ± 0.74	0.90
SDS BMI	−2.3 ± 0.28	−1.96 ± 0.34	0.001**	−2.37 ± 0.31	−2.36 ± 0.79	0.95
MUAC (cm)	14.76 ± 0.94	15.18 ± 0.84	0.005**	15.22 ± 0.8	15.52 ± 0.95	0.07
Biceps (mm)	3.912 ± 1.05	3.83 ± 1.124	0.56	3.78 ± 1.01	3.78 ± 1.18	0.57
Triceps (mm)	6.18 ± 1.26	6.4 ± 1.5	0.28	6.43 ± 1.59	6.43 ± 2.01	0.95
Mid abdomen (mm)	4.49 ± 1.49	4.71 ± 1.58	0.22	5.04 ± 1.6	5.15 ± 1.7	0.39
Sub scapular (mm)	4.58 ± 0.66	4.6 ± 0.77	0.5	4.73 ± 0.83	4.76 ± 0.85	0.7
Waist to hip ratio	0.85 ± 0.04	0.84 ± 0.04	0.09	0.85 ± 0.08	0.82 ± 0.06	0.07

*Note*: Values are presented as mean ± S.D.

*p < .05; **p < .01; ***p < .001.

### 3.3 Blood parameters analysis

The levels of total cholesterol (TC) (mg/dl) (*p* < .01) and fasting glucose (mg/dl) (*p* < .01) were significantly high in the LNS group after 4 weeks of supplementation as compared to the PLACEBO group (Table 4).

**TABLE 4:** Comparison of lipid parameters, insulin, and glucose between first and second trial days of the LNS group and the PLACEBO group

Parameters	LNS (*n* = 16)			PLACEBO (*n* = 16)		
	Day 1	Day 31	*p*‐Value	Day 1	Day 31	*p*‐Value
TC (mg/dl)	162.5 ± 21.72	186.08 ± 35.99	0.005**	159.02 ± 27.3	158.55 ± 22.42	0.96
TG (mg/dl)	84.53 ± 23.33	74.67 ± 23.95	0.21	88.38 ± 31.83	77.29 ± 26.26	0.20
HDL‐C (mg/dl)	56.13 ± 59.44	55.41 ± 16.71	0.96	52.33 ± 14.38	47.73 ± 12.31	0.20
LDL‐C (mg/dl)	118.6 ± 16.71	122.77 ± 27.95	0.52	115.01 ± 27.9	96.01 ± 23.64	0.06
VLDL‐C (mg/dl)	16.75 ± 4.62	14.89 ± 4.85	0.24	18.25 ± 5.94	14.81 ± 4.56	0.07
Insulin (ml U/L)	1.85 ± 1.21	2.04 ± 0.81	0.56	2.53 ± 1.49	2.78 ± 2.7	0.66
Glucose (mg/dl)	91.17 ± 10.79	98.62 ± 9.76	0.005*	99.89 ± 13.7	104.06 ± 10.69	0.26
HOMA (IR)	7.82 ± 5.76	8.99 ± 3.68	0.41	11.43 ± 7.05	13.24 ± 13.86	0.54

*Note*: Values are presented as mean ± S.D.

Abbreviations: HDL‐C, High‐density lipoprotein cholesterol; HOMA (IR), Homeostatic model assessment (Insulin resistance); LDL‐C, Low‐density lipoprotein cholesterol; TC, Total cholesterol; TG, Triglyceride; VLDL‐C, Very low‐density lipoprotein cholesterol.

*p < .05; **p < .01; ***p < .001.

### 3.4 Energy intake (EI) on trial days

#### 3.4.1 EI on first trial day

A significant increase in EI (*p* < .001), fats (*p* < .001), proteins (*p* < .001), and carbohydrates (*p* < .01) was observed in the LNS group. However, during breakfast and lunch, the total EI (*p* < .05) and carbohydrates intake (*p* < .01) was significantly lower in LNS. Similarly, when the sum of energy intake during breakfast and lunch was analyzed, energy intake (*p* < .01) and intake of proteins (*p* < .05), fats (*p* < .05), and carbohydrates (*p* < .01) of LNS was found to be significantly lower than the PLACEBO group. Also, a significant decrease was observed in only carbohydrates (*p* < .01) in the LNS group when the total EI was combined (Table 5).

**TABLE 5:** Comparison of energy intake between the LNS group and the PLACEBO group on first and second trial day

	1st trial Day				2nd trial day		
		LNS	PLACEBO	*p*‐Value	LNS	PLACEBO	*p*‐Value
Supplement	Energy (kcal)	280.31 ± 162.4	30.12 ± 20.9	<0.001***	257.18 ± 132.4	32.8 ± 24.7	<0.001***
	Proteins (g)	7.33 ± 4.26	1.82 ± 1.22	<0.001***	6.73 ± 3.46	1.98 ± 1.42	<0.001***
	Fats (g)	15.71 ± 9.1	0.18 ± 0.12	<0.001***	14.4 ± 7.4	0.21 ± 0.15	<0.001***
	CHO (g)	11.58 ± 6.74	4.65 ± 3.2	0.002**	10.58 ± 5.43	5.13 ± 3.7	0.002**
Breakfast	Energy (kcal)	289.9 ± 126.6	464.35 ± 244.9	0.017*	313.56 ± 123.3	417.18 ± 222.8	0.11
	Proteins (g)	12.03 ± 4.84	14.08 ± 8.65	0.41	12.07 ± 3.62	13.98 ± 7.34	0.36
	Fats (g)	15.65 ± 6.79	21.8 ± 12.01	0.08	17.4 ± 6.23	20.77 ± 10.79	0.09
	CHO (g)	26.7 ± 16.45	55.93 ± 30.24	0.002**	28.9 ± 16.6	45.8 ± 27.54	0.04*
Lunch	Energy (kcal)	212.6 ± 112.8	329.7 ± 137.5	0.01*	336.18 ± 247.1	333.68 ± 160.23	0.97
	Proteins (g)	4.7 ± 3.57	9.21 ± 5.64	0.14	7.58 ± 5.9	7.82 ± 3.89	0.89
	Fats (g)	4.7 ± 3.7	7.53 ± 4.07	0.05	7.8 ± 6.28	7.23 ± 3.15	0.72
	CHO (g)	39.9 ± 21.2	59.8 ± 24.1	0.02**	62.66 ± 45.3	61.7 ± 29.7	<0.001***
Breakfast + Lunch	Energy (kcal)	501.8 ± 187.7	793.93 ± 301.6	0.003**	644.8 ± 301.4	751.5 ± 318.9	0.34
	Proteins (g)	16.7 ± 6.6	23.71 ± 9.4	0.02*	19.6 ± 8.29	21.8 ± 9.6	0.49
	Fats (g)	19.17.84	29.4 ± 12.5	0.01*	25.2 ± 10.04	28.88 ± 12.8	0.38
	CHO (g)	66.7 ± 28.8	112.4 ± 49.2	0.003**	91.6 ± 48.6	151.2 ± 172.8	0.19
Breakfast + Lunch + Supplement	Energy (kcal)	782.2 ± 273.1	824.1 ± 312.4	0.69	902 ± 323.7	784.3 ± 323.14	0.31
	Proteins (g)	24.1 ± 9.2	25.5 ± 9.9	0.66	26.37 ± 8.6	23.8 ± 9.87	0.44
	Fats (g)	36.05 ± 14.9	29.5 ± 12.4	0.19	39.68 ± 10.9	28.7 ± 12.7	0.02*
	CHO (g)	78.2 ± 29.4	120.5 ± 47.1	0.005**	102.13 ± 49.2	112.6 ± 47.5	0.54

*Note*: Values are presented as mean ± S.D.

Abbreviations: CHO, Carbohydrate.

*p < .05; **p < .01; ***p < .001.

#### 3.4.2 EI on second trial day

After 4 weeks of supplementation, the energy intake (*p* < .001), proteins (*p* < .001), fats (*p* < .001) and carbohydrate (*p* < .01) was significantly high in the LNS group after provision of supplement. During breakfast (*p* < .05) and lunch (*p* < .001), only CHO intake was significantly lower in the LNS group. Also, a significant increase was found only in fats intake (*p* < .05) in the LNS group when total energy intake was calculated (Table 5).

### 3.5 Multiple pass 24‐ hour dietary recalls measurements

No significant difference was observed in energy intake and the macronutrient intake between LNS and PLACEBO groups before the 1st trial day. However, after 3 days of the first trial day, the total energy, proteins, and fats intake were significantly raised in the LNS group (*p* < .001) as compared to the PLACEBO group. Similarly, after 15 days of the first trial day, again the energy (*p* < .001), proteins (*p* < .01), and fats (*p* < .001) intake were significantly higher in the LNS group in comparison to the PLACEBO group (*p* < .001) (Table S1).

### 3.6 Appetite analysis

**FIGURE 3:**
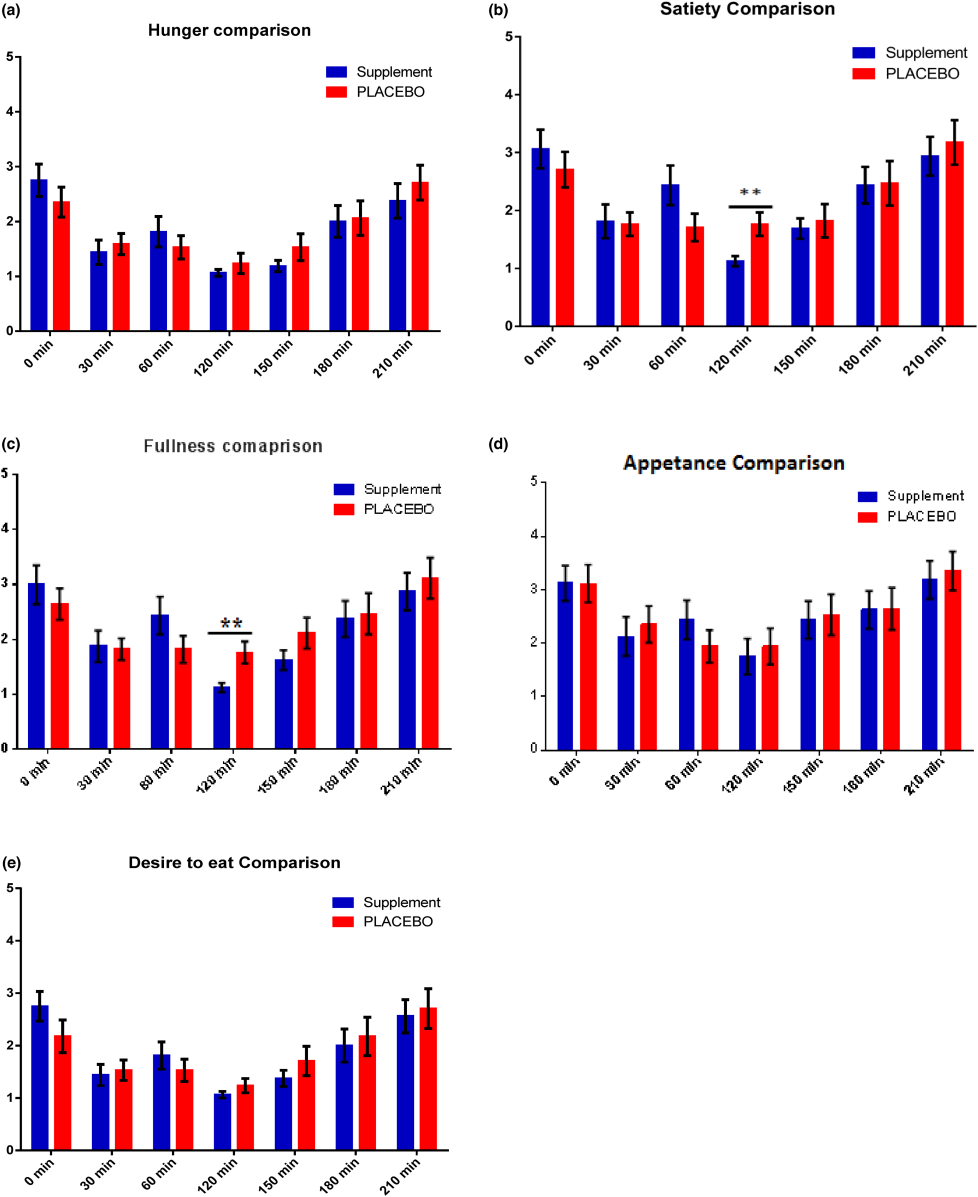
Bar charts showing the comparison of the (a) hunger (b) satiety (c) fullness (d) appetence, and (e) desire to eat responses between LNS and PLACEBO groups during fasted state, pre‐breakfast and pre‐lunch period at the 1st trial day. Significantly different (*p < .05, **p < .01, ****p < .001).

Appetite analysis showed that all appetite measures were not significantly different between the LNS group and the PLACEBO group. However, during pre‐lunch period (120–240 min) on first trial day, a significant increase (*p* < .01) was observed in summary response of satiety and fullness in the PLACEBO group as compared to the LNS group (Table S2, Figure 3b,c).

**FIGURE 4:**
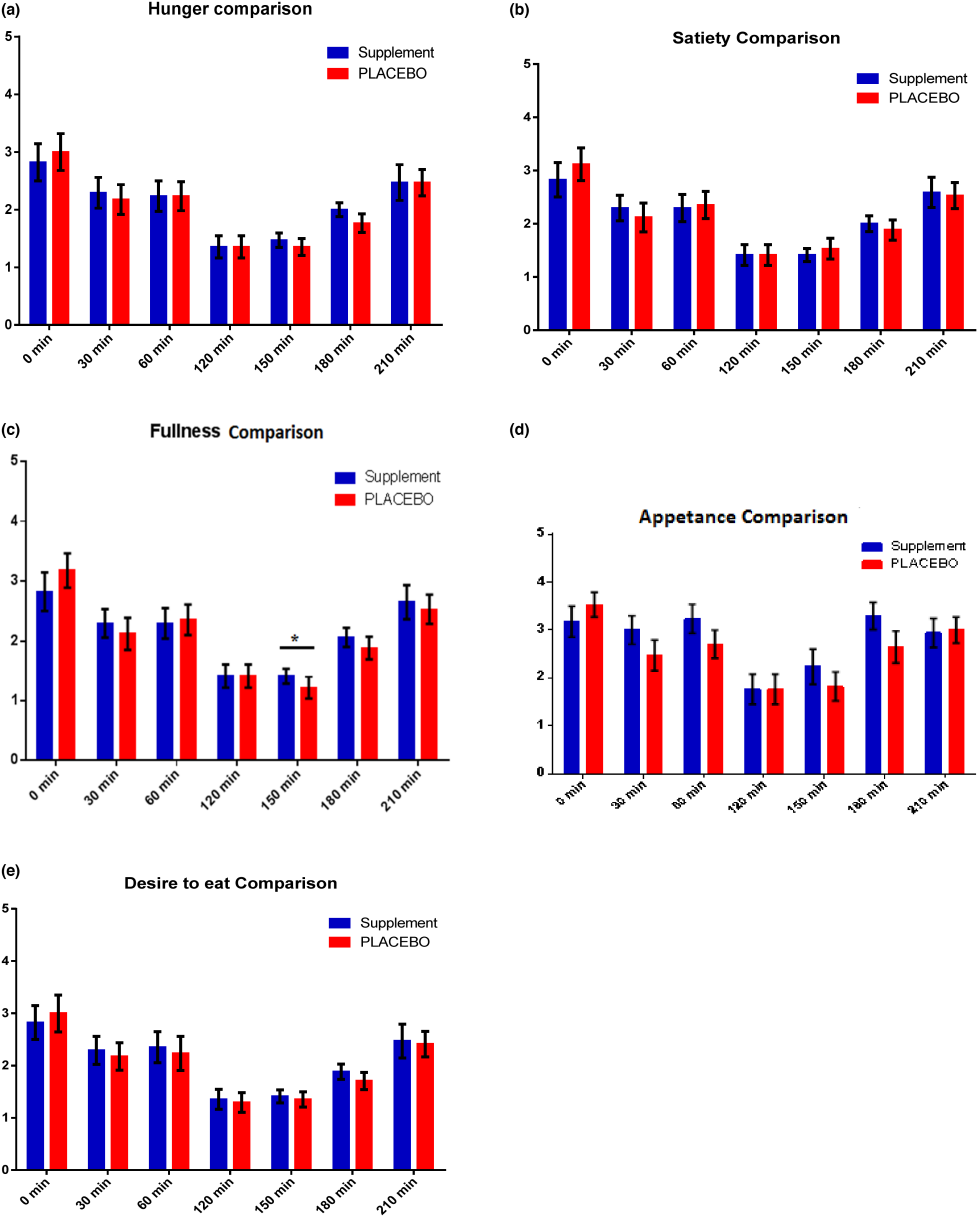
Bar charts showing the comparison of the (a) hunger (b) satiety (c) fullness (d) appetence, and (e) desire to eat responses between LNS and PLACEBO groups during fasted state, pre‐breakfast and pre‐lunch period at the second trial day. Significantly different (**p < .05, ***p < .01, ****p < .001).

Similarly, on second trial day again during pre‐lunch period (120–240 min), response of fullness was found significantly higher (*p* < .05) in the LNS group than in the PLACEBO group (Table S3, Figure 4c).

### 3.7 Hedonic scale analysis

Most participants liked the taste of supplements. Only 6.25% of children slightly disliked the taste of LNS and PLACEBO from both the groups (Table S4).

## 4 DISCUSSION

High prevalence of childhood malnutrition in developing countries has significantly raised the overall mortality rate and disease burden (Smith et al., 2003). Over time, different interventional/supplementary feeding programs are introduced (Medoua et al., 2016; Nikièma et al., 2014; Prado et al., 2016; Vanelli et al., 2014) to check the efficiency of nutritional supplements but there is scarcity of data on school‐going MAM children. Therefore, the current study investigated the effect of LNS on energy intake, appetite, and lipid profile of MAM children. We found a significant increase in total energy intake, body weight, BMI, MUAC, total cholesterol, and fasting glucose levels after 4 weeks of LNS. However, no significant changes in appetite responses were observed.

On first trial day, a significant decrease in energy intake during breakfast was observed in the LNS group after provision of supplement. Similar energy suppression was found during breakfast and dinner in other studies after the intake of supplement (Fatima, et al., 2015; Fatima, Gerasimidis, Wright, & Malkova, 2015). However, prolonged energy suppression was observed in our study which extended up to lunch in contrast to other two studies (Fatima, Gerasimidis, Wright, & Malkova, 2015; Fatima, et al., 2015). Similar prolonged energy suppression after the use of LNS was observed in a recent study on malnourished children (Nawab et al., 2022) which shows that energy is compensated due to intake of high caloric supplement. On second trial day, the total energy intake was higher in the LNS group than in the PLACEBO group during breakfast and lunch. Similarly, no energy suppression is reported previously in other studies (Fatima, Gerasimidis, Wright, & Malkova, 2015; Nawab et al., 2022). This suggests that children can accommodate the feeding of supplement if it is given on daily basis and energy consumption is not reduced.

We also checked the habitual EI of participants using Multiple‐pass 24‐hour dietary recalls 3 days before the first trial, 3 days after the first trial and then 15 days after the first trial day. Energy intake at the baseline showed no significant difference however, a significant increase was found in the LNS group in comparison to the PLACEBO group (*p* < .001) after three and 15 days of LNS. Similar results were observed in previous studies where supplementation caused significant increase in energy intake of participants (Fatima, et al., 2015; Huynh et al., 2015; Parsons et al., 2017; Samadi et al., 2016).

In this study, we also found that the effect of the extra energy (535 kcal/day) provided by supplement on weight gain of participants was quite noticeable after 4 weeks of supplementation; however, it was much lower than expected. The average increase in the weight of LNS participants was 0.6 kg instead of expected 2 kg. It can be explained in terms of energy compensation due to supplement provision. Similar results were found in some other studies on undernourished children where the observed weight gain was lower than predicted values due to lowered habitual energy intake after using supplementation on daily basis (Fatima et al., 2018; Lazzerini et al., 2013; Lenters et al., 2013). Appetite assessment revealed suppression in breakfast in LNS but the appetite was not affected suggesting there was a short‐term appetite suppression. On first trial day of our study, the PLACEBO group felt much satisfied and full as compared to the LNS group in pre‐lunch period. This is also reported previously (Dossa et al., 2001, 2002). One of the reason for this short‐term suppression could be the limited time span of supplement administration, breakfast, and lunch. This sort of suppression which was short‐lived was also observed in underweight females when supplement was taken in the morning (Fatima, et al., 2015).

Similarly, on last day of trial, appetite was not affected during pre‐breakfast period; however, a significant rise was observed in fullness score of the LNS group during pre‐lunch period. This indicates adaptation to the supplementation however, the short‐term effect on appetite was still preserved. These results are in contrast to findings by Shakur et al., 2009, with a significant increase in appetite of children (Shakur et al., 2009). However, as soon as the participants discontinued the supplement, their poor appetite appeared again (Shakur et al., 2009). Further investigation on the hormones regulating appetite patterns will be helpful in this regard.

Improvements in anthropometric parameters are also observed in previous studies (Ackatia‐Armah et al., 2015; Dewey et al., 2021; Fabiansen et al., 2017). A significant improvement in weight gain of MAM children (Collins, 2004) and malnourished gastrointestinal patients was observed after the use of supplementation (Kennedy et al., 2015). Likewise, previous literature shows that use of LNS significantly improved BMI as well as MUAC in malnourished children (Dewey et al., 2021; Fabiansen et al., 2017). No change in the height was observed after 4 weeks of supplementation as it was for short duration. Similar to our findings short‐term supplementation did not affect height or skin fold thickness to a significant level in malnourished adults and children (World Health Organization, 2007).

Glucose and insulin both play important roles in appetite regulation (Woods et al., 2006). In our study, a significant increase in baseline glucose levels was observed after 4 weeks of supplementation in the LNS group. The same was observed by Fatima et al. (Fatima, et al., 2015) in lean women after the use of high‐energy nutritional supplement drinks (HENSDs). However, in contrast to our results, a study found no significant difference in glucose levels after using supplements for 15 days (Althuis et al., 2002).

We found no significant difference in insulin levels after LNS use. Similar to our results, another study on healthy individuals found no significant difference in insulin levels after the administration of supplements (Althuis et al., 2002). However, in contrast to our results, insulin levels significantly raised in a trial performed on thin women after using HENSDs (Fatima, et al., 2015). These findings suggest that the way of metabolization of insulin might vary between women and children.

We compared the data on lipid profile before and after taking supplementation for 4 weeks. No significant difference was found in the PLACEBO group among lipid profile parameters on day 1st and 31st. However, we observed a significant increase in total cholesterol (TC) levels in the LNS group. In contrast to our study, a significant decrease in TC and HDL levels was observed after supplement use by individuals from different ethnic groups (Dodin et al., 2005). The reason for significantly higher levels of TC in our study might be related to ethnicity as lipid profile is multifactorial (Tejada et al., 1968). Furthermore, we found significant increase in fat content in the LNS group. The LNS used in our study also contains high‐fat content (Table 1). Similarly, high TC levels were found in previous studies, where high‐fat diet was used (Enos et al., 2013; Lorenzen & Astrup, 2011).

Strengths of this study were that EI data were analyzed by three researchers individually to enhance the accuracy of results. Similarly, habitual EI was obtained using Multiple pass 24‐hour dietary recalls to reduce misreporting biasness. Also, to maintain good compliance, supplements were provided on daily basis and empty sachets were collected by the main researchers on the same day. Limitation of this study was that mechanism of appetite suppression and appetite hormones like leptin and ghrelin could not be checked.

After our investigation, we recommend long‐term studies to evaluate the effects of appetite hormones and appetite pattern in malnourished and healthy children. We also recommend nutritional counseling along with nutritional programs which might prove to be an effective strategy for tackling nutritional problems in children.

## 5 CONCLUSION

LNS causes improvement in nutritional outcomes of mild‐to‐moderate underweight children. It has proven to be effective in increasing the overall energy intake but it has also reduced the regular dietary intake of children due to which the observed weight gain was less than expected. Moreover, the use of LNS only for 1 month induces hyperglycemia and hyperlipidemia.

## FUNDING INFORMATION

The current research was partially funded to the research scholar Aqsa Zubair (MPhil scholar) by the Organization of Research Innovation and commercialization (ORIC) of Khyber Medical University.

## CONFLICT OF INTERESTS

The authors declared no conflict of interests. All authors approved the final manuscript.

## Supporting information


**Supplementary Table 1** Comparison of multiple pass 24‐hours dietary recalls during follow‐up between the LNS group and the PLACEBO group. Values are presented as Mean ± S.D.
**Supplementary Table 2.** Appetite comparison on first trial day between the LNS group and the PLACEBO group. Values are presented as Mean ± S.D.
**Supplementary Table 3.** Appetite comparison on second trial day between the LNS group and the PLACEBO group. Values are presented as Mean ± S.D.
**Supplementary Table 4.** Comparison of taste acceptability between the LNS group and the PLACEBO group.

## Data Availability

Available from the corresponding author on reasonable request.
