# Supplementary material for: Effects of Oral Lipid‐Based nutritional supplements on appetite, energy intake, and lipid profile of moderately underweight children
Source: Food Sci Nutr. 2022 Dec 18;11(2):903–16. doi: 10.1002/fsn3.3125 (PMC9922128; doi:10.1002/fsn3.3125)
Supplement: Supplementary file 1 — Supplementary Table 1 Comparison of multiple pass 24‐hours dietary recalls during follow‐up between the LNS group and the PLACEBO group. Values are presented as Mean ± S.D. Supplementary Table 2. Appetite comparison on first trial day between the LNS group and the PLACEBO group. Values are presented as Mean ± S.D. Supplementary Table 3. Appetite comparison on second trial day between the LNS group and the PLACEBO group. Values are presented as Mean ± S.D. Supplementary Table 4. Comparison of taste acceptability between the LNS group and the PLACEBO group. [file FSN3-11-903-s001.docx]

**Supplementary Table 1** Comparison of multiple pass 24-hours dietary recalls during follow-up between LNS group and PLACEBO group. Values are presented as Mean ± S.D.

|  | **LNS (n=16)** | **PLACEBO (n=16)** | **p-Value** |
| --- | --- | --- | --- |
| **Three days before trial** | | | |
| Energy (kcal)  Proteins (g)  Fats (g)  †CHO (g) | 702 ± 128.6  21.5 ± 3.9  28.3 ± 7.8  91.7 ±15.9 | 629 ± 120  19.7 ± 5.3  24.5 ± 7.5  87.8 ± 13.2 | 0.11  0.27  0.2  0.46 |
| **Three days after trial** | | | |
| Energy (kcal)  Proteins (g)  Fats (g)  CHO (g) | 1036.2 ± 131.6  31.3 ± 5.9  48.4 ± 8.9  96.2 ± 13.5 | 678.5 ± 157.4  23.1 ± 5.8  23.9 ± 8.6  95.5 ± 20.1 | <0.001*** <0.001***  <0.001***  0.9 |
| **Three days after fifteen days of trial** | | | |
| Energy (kcal)  Proteins (g)  Fats (g)  CHO (g) | 1024.4 ± 171.2  30.3 ± 6.7  45.2 ± 8.3  106 ± 26.4 | 726.8 ± 145.4  23.7 ± 4.7  26.4 ± 7.7  102.8 ± 20.3 | <0.001***  0.003**  < 0.001***  0.7 |

Significantly different * p < 0.05, ** p < 0.01, *** p < 0.001

† Carbohydrate

**Supplementary Table 2** Appetite comparison on first trial day between LNS group and PLACEBO group. Values are presented as Mean ± S.D.

| **DAY 1** | | | | | | | | | | | | | | | |
| --- | --- | --- | --- | --- | --- | --- | --- | --- | --- | --- | --- | --- | --- | --- | --- |
|  | **Hunger** | | | **Satiety** | | | **Fullness** | | | **Appetence** | | | **Desire to eat** | | |
|  | **LNS** | **PLACEBO** | **p** | **LNS** | **PLACEBO** | **p** | **LNS** | **PLACEBO** | **p** | **LNS** | **PLACEBO** | **p** | **LNS** | **PLACEBO** | **p** |
| 0 min | 2.75 ±1.18 | 2.43 ±1.09 | 0.44 | 3.06± 1.34 | 2.81±1.22 | 0.58 | 3 ±1.4 | 2.75 ±1.13 | 0.58 | 3.13±1.31 | 3.25 ±1.39 | 0.79 | 2.75± 1.12 | 2.25 ±1.29 | 0.25 |
| 30 min | 1.44±0.89 | 1.63 ±0.81 | 0.53 | 1.81± 1.17 | 1.81± 0.83 | 1 | 1.87± 1.15 | 1.87± 0.81 | 1 | 2.12± 1.45 | 2.43±1.41 | 0.54 | 1.44± 0.81 | 1.56 ±0.82 | 0.67 |
| 60  Min | 1.8125 ±1.11 | 1.57± 0.89 | 0.48 | 2.4±1.36 | 1.7±5 1 | 0.11 | 2.43± 1.36 | 1.87 ±1.02 | 0.19 | 2.43± 1.46 | 2 ±1.26 | 0.37 | 1.81± 1.05 | 1.56 ±0.89 | 0.47 |
| 120  Min | 1.06± 0.25 | 1.06± 0.25 | 1 | 1.12± 0.34 | 1.63± 0.62 | 0.008** | 1.12± 0.34 | 1.63± 0.62 | 0.008** | 1.7±1.34 | 1.94± 1.44 | 0.71 | 1.06± 0.25 | 1.13 ±0.34 | 0.56 |
| 150  Min | 1.19 ±0.4 | 1.37± 0.8 | 0.41 | 1.69± 0.7 | 1.689± 1.08 | 1 | 1.62± 0.73 | 2± 1.09 | 0.26 | 2.44± 1.4 | 2.56± 1.63 | 0.81 | 1.37± 0.62 | 1.63 ±1.15 | 0.45 |
| 180  Min | 2± 1.15 | 1.87± 1.09 | 0.75 | 2.44± 1.26 | 2.3 ±1.49 | 0.79 | 2.37± 1.31 | 2.31± 1.45 | 0.89 | 2.62± 1.41 | 2.5 ±1.55 | 0.81 | 2± 1.26 | 2± 1.37 | 1 |
| 210  Min | 2.38± 1.26 | 2.56± 1.21 | 0.67 | 2.94± 1.34 | 3.06± 1.57 | 0.81 | 2.87± 1.36 | 3 ±1.51 | 0.81 | 3.18± 1.42 | 3.25± 1.48 | 0.9 | 2.56± 1.26 | 2.5± 1.5 | 1 |

Significantly different * p < 0.05, ** p < 0.01, *** p < 0.001

**Supplementary Table 3** Appetite comparison on second trial day between LNS group and PLACEBO group. Values are presented as Mean ± S.D.

| **DAY 31** | | | | | | | | | | | | | | | |
| --- | --- | --- | --- | --- | --- | --- | --- | --- | --- | --- | --- | --- | --- | --- | --- |
|  | **Hunger** | | | **Satiety** | | | **Fullness** | | | **Appetite** | | | **Desire to eat** | | |
|  | **LNS** | **PLACEBO** | **p** | **LNS** | **PLACEBO** | **p** | **LNS** | **PLACEBO** | **p** | **LNS** | **PLACEBO** | **p** | **LNS** | **PLACEBO** | **p** |
| 0 min | 2.69±  1.25 | 3.06±  1.34 | 0.42 | 2.69±  1.25 | 3.19±  1.28 | 0.27 | 3.13±  1.31 | 3.25±  1.18 | 0.78 | 3.06±  1.29 | 3.50±  1.10 | 0.31 | 2.69±  1.25 | 3.06±  1.48 | 0.45 |
| 30 min | 2.31±  1.14 | 2.19±  1.11 | 0.76 | 2.31±  1.01 | 2.13±  1.15 | 0.63 | 2.13±  1.45 | 2.13±  1.15 | 1.00 | 2.94±  1.24 | 2.38±  1.31 | 0.22 | 2.31±  1.14 | 2.19±  1.11 | 0.76 |
| 60 min | 2.25±  1.13 | 2.19±  1.05 | 0.87 | 2.31±  1.08 | 2.31±  1.08 | 1.00 | 2.44±  1.46 | 2.31±  1.08 | 0.78 | 3.19±  1.28 | 2.69±  1.25 | 0.27 | 2.38±  1.26 | 2.19±  1.38 | 0.69 |
| 120 min | 1.31±  0.79 | 1.31±  0.79 | 1.00 | 1.38±  0.81 | 1.38±  0.81 | 1.00 | 1.75±  1.34 | 1.38±  0.81 | 0.35 | 1.63±  1.20 | 1.63±  1.20 | 1.00 | 1.31±  0.79 | 1.25±  0.77 | 0.82 |
| 150 min | 1.44±  0.51 | 1.31±  0.60 | 0.53 | 1.38±  0.50 | 1.50±  0.82 | 0.61 | 2.44±  1.41 | 1.50±  0.82 | 0.03* | 2.13±  1.50 | 1.69±  1.14 | 0.36 | 1.38±  0.50 | 1.31±  0.60 | 0.75 |
| 180 min | 1.94±  0.44 | 1.75±  0.68 | 0.36 | 1.94±  0.57 | 1.88±  0.81 | 0.80 | 2.63±  1.41 | 1.88±  0.81 | 0.07 | 3.31±  1.20 | 2.56±  1.36 | 0.11 | 1.81±  0.54 | 1.69±  0.70 | 0.58 |
| 210 min | 2.38±  1.26 | 2.50±  0.97 | 0.75 | 2.50±  1.15 | 2.56±  1.03 | 0.87 | 3.19±  1.42 | 2.56±  1.03 | 0.17 | 3.00±  1.26 | 2.94±  1.12 | 0.88 | 2.38±  1.31 | 2.44±  1.03 | 0.88 |

Significantly different * p < 0.05, ** p < 0.01, *** p < 0.001

**Supplementary Table 4** Comparison of taste acceptability between LNS group and PLACEBO group.

|  | LNS (n=16) | | PLACEBO (n=16) | |
| --- | --- | --- | --- | --- |
|  | **n (%)** | **p-value** | **n (%)** | **p-value** |
| Extremely tasty | 2 (12.50) | 0.903 | 1 (6.25) | 0.848 |
| Very tasty | 4 (25.00) |  | 5 (31.25) |  |
| Moderately tasty | 5 (31.25) |  | 4 (25.00) |  |
| Slightly tasty | 4 (25.00) |  | 2 (12.50) |  |
| Neither like nor dislike | - |  | 3 (18.75) |  |
| Dislike slightly | 1 (6.25) |  | 1 (6.25) |  |
| Dislike moderately | - |  | - |  |
| Dislike very much | - |  | - |  |
| Dislike extremely | - |  | - |  |

Significantly different * p < 0.05, ** p < 0.01, *** p < 0.001
